# Antigen-Specific Single B Cell Sorting and Monoclonal Antibody Cloning in Guinea Pigs

**DOI:** 10.3389/fmicb.2019.00672

**Published:** 2019-04-23

**Authors:** Lin Lei, Karen Tran, Yimeng Wang, James J. Steinhardt, Yongli Xiao, Chi-I Chiang, Richard T. Wyatt, Yuxing Li

**Affiliations:** ^1^Institute for Bioscience and Biotechnology Research, University of Maryland, Rockville, MD, United States; ^2^IAVI Neutralizing Antibody Center, The Scripps Research Institute, La Jolla, CA, United States; ^3^Laboratory of Infectious Diseases, National Institute of Allergy and Infectious Diseases, National Institutes of Health, Bethesda, MD, United States; ^4^Department of Immunology and Microbiology, The Scripps Research Institute, La Jolla, CA, United States; ^5^Department of Microbiology and Immunology, University of Maryland School of Medicine, Baltimore, MD, United States

**Keywords:** guinea pig, B cells, HIV-1, envelope glycoproteins, immunization, single cell RT-PCR, monoclonal antibodies

## Abstract

Here, we have established an antigen-specific single B cell sorting and monoclonal antibody (mAb) cloning platform for analyzing immunization- or viral infection-elicited antibody response at the clonal level in guinea pigs. We stained the peripheral blood mononuclear cells (PBMCs) from a guinea pig immunized with HIV-1 envelope glycoprotein trimer mimic (BG505 SOSIP), using anti-guinea pig IgG and IgM fluorochrome conjugates, along with fluorochrome-conjugated BG505 SOSIP trimer as antigen (Ag) probe to sort for Ag-specific IgG^hi^ IgM^lo^ B cells at single cell density. We then designed a set of guinea pig immunoglobulin (Ig) gene-specific primers to amplify cDNAs encoding B cell receptor variable regions [V(D)J segments] from the sorted Ag-specific B cells. B cell V(D)J sequences were verified by sequencing and annotated by IgBLAST, followed by cloning into Ig heavy- and light-chain expression vectors containing human IgG1 constant regions and co-transfection into 293F cells to reconstitute full-length antibodies in a guinea pig-human chimeric IgG1 format. Of 88 antigen-specific B cells isolated, we recovered 24 (27%) cells with native-paired heavy and light chains. Furthermore, 85% of the expressed recombinant mAbs bind positively to the antigen probe by enzyme-linked immunosorbent and/or BioLayer Interferometry assays, while five mAbs from four clonal lineages neutralize the HIV-1 tier 1 virus ZM109. In summary, by coupling Ag-specific single B cell sorting with gene-specific single cell RT-PCR, our method exhibits high efficiency and accuracy, which will facilitate future efforts in isolating mAbs and analyzing B cell responses to infections or immunizations in the guinea pig model.

## Introduction

Antibodies are Y-shaped globular proteins, namely Ig, that are produced by the immune system in most vertebrates (Boehm, 2012). The Ig molecules typically consist of two identical heavy chains (IGH) and two light chains (IGL or IGK) (Schroeder et al., 2010). They are either secreted by or presented on the surface of B lymphocytes fulfilling crucial functions during the course of humoral immune responses to prevent or combat infections (Moser and Leo, 2010). The antigen-binding specificity is mainly determined by the antibody variable region, which is assembled from germline variable (V), diversity (D) for heavy chain, and joining (J) gene segments (Yeap et al., 2015). The number of functional V, D, and J genes is limited across different species. However, the immune system has evolved a complex process to generate antibody repertoire with almost infinite diversity, mainly through random nucleotide insertions and deletions at V(D)J junctions, and somatic hypermutation (SHM) (Yeap et al., 2015).

As each antibody has precise specificity for a given antigen, mAbs are widely used in biological research, clinical diagnosis, and therapy (Clark, 1986). Various techniques have been developed to isolate mAbs from humans and immunized animals. Hybridomas and Epstein-Barr Virus (EBV) immortalized B cells are among the most commonly used platforms, however, the efficiency of each technology is relatively low, which compromises sampling the diversity of immune repertoire (Pasqualini and Arap, 2004; Kwakkenbos et al., 2016). While antibody display methodologies such as phage and yeast display libraries are widely adopted for mAb isolation, they are prone to generate biased repertoires and lose information of natural pairing (Saggy et al., 2012). Single B cell technologies have evolved rapidly in recent years (Wardemann et al., 2003; Tiller et al., 2008), and have been applied to isolate numerous bNAbs against HIV, Ebola, and influenza (Fu et al., 2016; McCoy and Burton, 2017; Zhao et al., 2017). Strategies to directly clone antibody sequences from single B cells for mouse, rabbit and macaque models have subsequently been developed to characterize immune responses at high resolutions (Tiller et al., 2009; Sundling et al., 2012; McCoy et al., 2016; Starkie et al., 2016).

The guinea pig is considered as the premier model in the study of infectious diseases (Padilla-Carlin et al., 2008). It shares many similarities to humans regarding symptoms and immune responses to infections and therapies (Tree et al., 2006; Padilla-Carlin et al., 2008). Additionally, unlike other small animal models such as the mouse model, the guinea pig model allows sampling significant blood volumes for downstream immunological analysis. Despite these advantages, the immune response of guinea pig model is still relatively understudied, which is largely due to the shortage of guinea pig-specific immune reagents and the lack of basic knowledge about Ig genes (Tree et al., 2006). To delineate the epitope specificity of B cell responses in guinea pigs, we established an antigen-specific single B cell sorting and mAb-cloning platform for the guinea pig model. By using newly designed guinea pig Ig gene-specific primers, we directly cloned and expressed antigen-specific mAbs from B cells isolated from guinea pigs immunized with HIV-1 envelope glycoprotein (Env) vaccine candidate BG505 SOSIP by FACS-based single cell sorting. This platform allows us to delineate antigen-specific antibody responses in guinea pigs at the clonal level for better understanding the immunogenicity of vaccine candidates and the effect of immunization strategies. Furthermore, this methodology is applicable for isolating/developing essential research reagents and therapeutic mAbs from guinea pigs in the future.

## Materials and Methods

### Animal Immunization and Sampling

**FIGURE 1:**
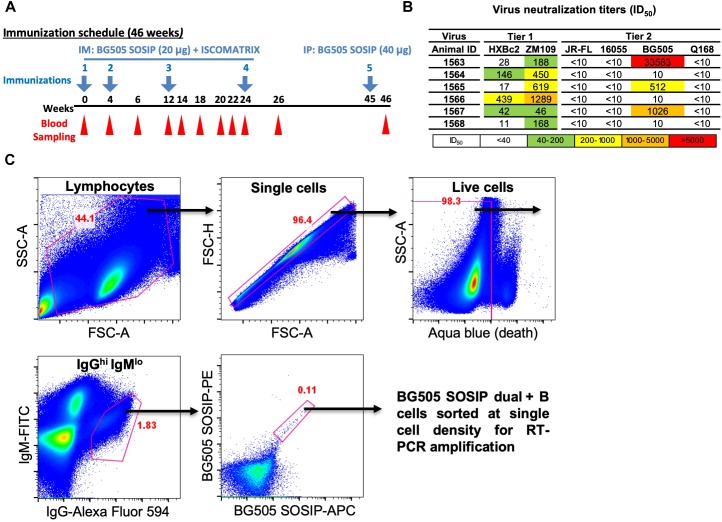
Isolation of vaccine-induced antigen-specific guinea pig B cells. **(A)** Guinea pigs (*n* = 6) were immunized at week 0, 4, 12, and 24 with BG505 SOSIP formulated in ISCOMATRIX adjuvant via intramuscular (IM) route. Serum sampling was performed at weeks indicated in the scheme. On week 45, BG505 SOSIP was injected by intraperitoneal (IP) route followed by termination bleed on week 46 and collection of spleens for splenocytes. **(B)** Neutralization ID_50_ titers (reciprocal serum dilution factor) of plasma collected at week 26 from guinea pigs against a panel of tier 1 and tier 2 viruses using the TZM-bl pseudovirus assay. The data are representative of at least two independent experiments. **(C)** Single B cell isolation was performed in an antigen-selective manner by multicolor fluorescenceactivated cell sorting (FACS). Peripheral blood mononuclear cells (PBMCs) from guinea pig 1567 on week 46 were stained by a cocktail of fluorochrome-conjugated antibodies and antigens for identifying IgG^hi^ IgM^lo^ B cell subpopulations with dual positive binding to BG505 SOSIP trimers to minimize non-specific antigen probe binding.

The guinea pig used in this study, designated as 1567, was immunized in a previous study (Feng et al., 2016). Briefly, along with another five guinea pigs in the same group, animal 1567 was immunized four times at weeks 0, 4, 12, and 24 with HIV-1 Env trimer BG505 SOSIP formulated in ISOMATRIX adjuvant. Blood samples were harvested 2 weeks after each immunization with the terminal bleed on week 46 to prepare for PBMC and sera for downstream analysis (Feng et al., 2016; Figure 1A,B). Four days prior to the termination, an inoculation of 40 μg of Env trimer BG505 SOSIP in the absence of adjuvant was administered by intraperitoneal injection (IP) route. The PBMCs from whole blood were further purified by density gradient centrifugation with Ficoll-Paque PLUS (GE Healthcare). After washing by PBS, cells were resuspended and frozen gradually in Bambanker media (Wako Chemicals) at -80°C followed by storage in liquid nitrogen prior to the staining and sorting experiment. The animal study was carried out at Covance with the protocol approved by the Covance Institutional Animal Care and Use Committee (IACUC, protocol #0138-14).

### Isolation of Single Guinea Pig B Cells by Fluorescence-Activated Cell Sorting (FACS)

Guinea pig PBMCs were thawed and re-suspended in 10 ml of pre-warmed RPMI 1640 medium (Gibco) supplemented with 10% FBS (Gibco) (R10) and 10 μl of DNase I (Roche). The cells were washed and re-suspended with 45 μl of pre-chilled phosphate-buffered saline (PBS). Five microliters of 40-fold water-diluted Live/dead fixable aqua dead stain (Invitrogen) was added to the cells followed by incubation in the dark at 4°C for 10 min. The cells were further stained by adding 50 μl of antibody cocktail in R10 medium containing anti-guinea pig IgM-FITC (100-fold dilution, Antibodies-online, ABIN457754), anti-guinea pig IgG-Alexa Fluor 594 (100-fold dilution, Jackson ImmunoResearch, 116790), and biotin-labeled HIV-1 Env trimer BG505 SOSIP conjugated with streptavidin-PE (Invitrogen) and streptavidin-APC (Invitrogen), respectively, at 4 μg/ml as described previously (Wu et al., 2010). The cell and antibody cocktail mixture was incubated in the dark at 4°C for 1 h. After staining, the cells were washed and re-suspended in 0.5 ml of pre-chilled R10 medium and passed through a 70 μm cell strainer (BD Biosciences) prior to cell sorting. Three microliters of Dynabeads^TM^ Protein G (Invitrogen) stained with the same volume of anti-guinea pig IgM-FITC and anti-guinea pig IgG-Alexa Fluor 594, respectively, as well as 20 μl of biotin bead (Spherotech, TP-30-5) stained with 0.1 μl of streptavidin-PE and streptavidin-APC, respectively, in a total volume of 100 μl at room temperature for 20 min, were used for compensation.

Antigen-specific single B cells were identified and sorted by a FACS Aria III cell sorter (BD Biosciences) at single cell density into 96-well PCR plates containing 20 μl of lysis buffer as previously described (Sundling et al., 2012). A representative example of FACS gating strategy used for identifying HIV Ag BG505 SOSIP-dual positive single B cells is shown in Figure 1C. In brief, after the gating of lymphocytes (SSC-A vs. FSC-A) and singlets (FSC-H vs. FSC-A), live cells were identified by the negative aqua blue staining phenotype. Antigen-specific IgG^hi^ B cells were then determined as IgG^hi^ IgM^lo^ and dual positive (PE^+^ APC^+^) for BG505 SOSIP probes. Percentage of gated cells in their parental cell population is shown in red (Figure 1C and Table 1).

**Table 1:** Statistic properties of the BG505-specific B cell sorting and Ig cloning.

	Guinea
Animal	pig #1567
Total PBMCs	10,050,000
Total Lymphocytes	4,429,926
Total Lymphocytes %	44.1
Total single cells	4,272,286
Total single cells %	96.4
Total live cells	4,199,193
Total live cells %	98.3
Total IgG^hi^ IgM^lo^ class-switched B cell	76,545
Total IgG^hi^ IgM^lo^ class-switched B cell %	1.82
Total IgG^hi^ IgM^lo^ BG505 SOSIP dual + class-switched B cell	81
Total IgG^hi^ IgM^lo^ BG505 SOSIP dual + class-switched B cell %	0.11
Sorted cells	88
Sorted cells with paired VH and VK/VL	24
Expressed mAbs	20
Clonal lineage of expressed mAbs	9
BG505 SOSIP + mAbs (assessed by ELISA)	16
Sorting precision [(GP+mAbs/Expressed mAbs)^∗^100], assessed by ELISA	80%
BG505 SOSIP + mAbs (assessed by BLI)	17
Sorting precision [(GP+mAbs/Expressed mAbs)^∗^100], assessed by BLI	85%
Ag-specific mAbs (assessed by ELISA, BLI, and clonal lineage analysis)	20
Precision of Ag-specific mAbs (assessed by ELISA, BLI, and clonal lineage analysis)	100%


### Guinea Pig Ig Gene-Specific Single Cell RT-PCR

We first performed reverse transcription (RT) to convert mRNA to cDNA with the sorted single B cells. We thawed the cells in lysis buffer followed by the addition of 450 ng random hexamers (Gene Link), 2 μl 10 mM dNTP (Sigma), 200 U Superscript III (Invitrogen) to a 26 μl final reaction volume. The RT program was set as the following: 10 min at 42°C, 10 min at 25°C, 60 min at 50°C, 5 min at 94°C, followed by held at 4°C.

**FIGURE 2:**
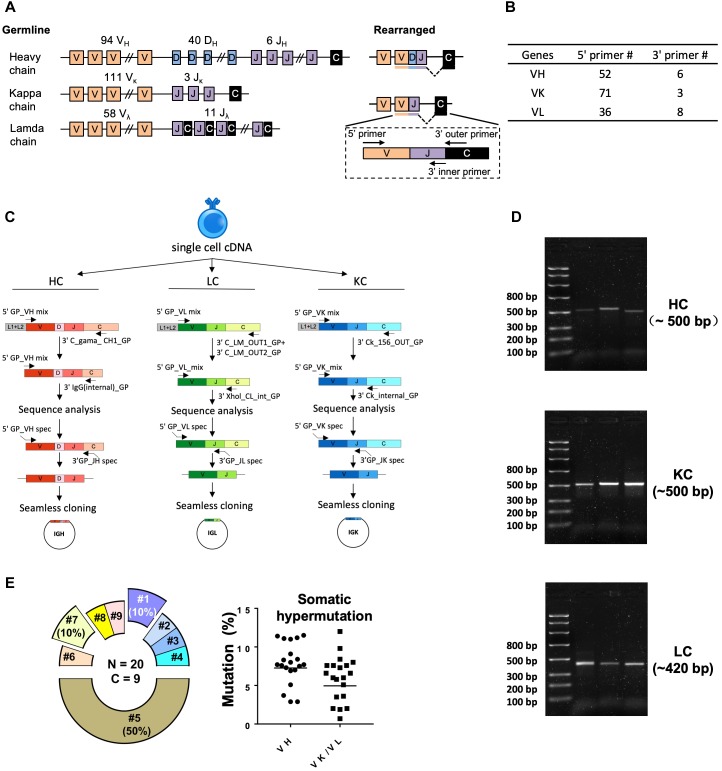
Single cell RT-PCR to amplify antigen-specific guinea pig B cell IGH, IGL, and IGK transcripts. **(A)** Guinea pig heavy and light chain gene organization and primer design. Antibody repertoire diversity is primarily determined by the somatic recombination of variable (V), diversity (D), and joining (J) gene segments, followed by the random non-templated nucleotides insertions in the V-D-J or V-J hotspots. Numbers of functional V, D, and J gene segments identified for heavy and light chains are indicated. **(B)** Numbers of primers designed for VH, VK, and VL amplification. **(C)** Immunoglobulin gene amplification by semi-nested PCR from single cell cDNA resulted from random-hexamer primed reverse transcription. The 1st PCRs were performed with 5′ forward primer mixtures and 3′ reverse primers specific for the heavy- or light-chain constant regions. The 2nd PCRs were performed with the same set of 5′ primers and 3′ reverse primers specific for the constant regions more adjacent to the J gene segments. The 2nd PCR products were sequenced to determine the V and J primers for seamless cloning PCR. Cloning PCR products were assembled with antibody heavy and light chain expression vectors. **(D)** Representative 2% gel electrophoresis patterns of the 2nd PCR products of heavy chain (HC, ∼500 bp), kappa chain (KC, ∼500 bp), and lambda chain (LC, ∼420 bp). **(E)** Genetic analysis of the sorted Ag-specific B cells with paired heavy- and light-chains. (Left) Clonal lineage analysis of the sorted putative Ag-specific mAb variable sequences. N, total number of sorted cells; C, total number of clonal lineages, which is determined by the following criteria: B cell clones with the same Ig V, J gene usage and identical CDR3 length with CDR3 nucleotide sequence homology >80% belong to the same clonal lineage and are likely derived from one naive B cell precursor. Each slice of the pie chart represents one clonal lineage. Clonal lineages (#1, #5, and #7) with multiple members are displayed as exploded slices. (Right) Somatic hypermutation levels of the Ag-specific B cell Ig variable regions (VH and VK/VL) were calculated as percentage of nucleotide sequence divergence from germline V gene sequences.

To amplify Ig encoding genes from the cDNA, we designed primers for semi-nested PCR reaction based on the guinea pig Ig gene segments recently identified (Guo et al., 2012; Figure 2 and Tables 2–4). The 5′ forward primers were designed to anneal to the 5′ end of the framework 1 (FR1) regions in V-gene segments. The 3′ reverse primers are situated in the constant region, with the 3′ inner primers for the 2nd PCR closer to J genes than the 3′ outer primers for the 1st PCR reactions (Figure 2C). The 1st PCR reaction was performed in a 50 μl reaction mixture consisting of 5 μl of cDNA, 5 μl of 10× PCR Buffer (Qiagen), 1 μl of 25 mM MgCl_2_ (Qiagen), 1 μl of 10 mM dNTPs (Sigma), 2 Unites of HotStar Taq Plus (Qiagen), 5 μl of 25 μM 5′ primer mixtures, and 1 μl of 25 μM 3′ outer primers. The 2nd PCR reaction mixture consisted of 2.5 μl of the same 5′ forward primer mixtures (25 μM) as in the 1st PCR with 0.5 μl of 25 μM 3′ inner primers as reverse primers (Figure 2C), and 5 μl 5× Q-solution without MgCl_2_ in 25 μl of volume. All the 5′ primers used for each heavy (VH), lambda (VL), and kappa (VK) chain amplification were stored at 25 μM and mixed in equal volume prior to the PCR reactions. All semi-nested PCRs were incubated at 94°C for 5 min followed by 50 cycles of 94°C for 30 s, 50°C for 45 s, and 72°C for 1 min with a final elongation at 72°C for 10 min before cooling to 4°C. The PCR products were evaluated on 2% 96-well E Gels (Life Technologies). Wells with expected sizes approximately 500 and 420 bp for heavy/kappa chains and lambda chain, respectively, were identified followed by PCR product purification and sequencing using downstream 3′ inner primers. The PCR primers for heavy, lambda, and kappa chains are described in Tables 2–4, respectively.

**Table 2:** Ig heavy PCR primer.

Single cell PCR	Primer	Sequence (5′–3′)	V gene segment
Heavy Chain Forward primers	5′GP_VH#1	GAGGAGCAACTGGTGGAGTCC	VH3-157, VH3-139
	5′GP_VH#2	CAGTTGCAGCTGCAGGAGTCA	VH1-14
	5′GP_VH#3	CAGGTGCAGCTGCAGGAGTCG	VH1-120, VH1-115, VH1-58
	5′GP_VH#4	CAGGTGCAGCTGCAGGAGTTG	VH1-56
	5′GP_VH#5	GAAGTACAGCTCACACAATCT	VH3-184
	5′GP_VH#6	GAGGTGCAGCTCGTGGAGTCT	VH3-170, VH3-167
	5′GP_VH#7	CAGGTTCAGCTGCAGGAGTCG	VH1-66, VH1-75
	5′GP_VH#8	CAGGTTCAGCTGCAGGAGTCA	VH1-1
	5′GP_VH#9	GAGGAGCAGCTGGTAGAGTCC	VH3-63, VH3-3
	5′GP_VH#10	GAGGTGCAGCTGGTGGAGTCT	VH3-35
	5′GP_VH#11	GAGGTGCAGCTGATGGAGTCC	VH3-230
	5′GP_VH#12	CAGGTGCAGCTACAGGAGTCG	VH1-95
	5′GP_VH#13	GAAGAGCAGCTGGTGGAGTCT	VH3-80
	5′GP_VH#14	GAGCCGCAGCTGGTGGAGTCA	VH3-194
	5′GP_VH#15	GAAGTACAGCTCACACAGTCT	VH3-201
	5′GP_VH#16	GAAGTGCAGCTCGTGGAGTCC	VH3-262
	5′GP_VH#17	GAAGTGCAACTCGTGGAGTCC	VH3-126
	5′GP_VH#18	GGTGCAGCTGGTGGAGTCCGG	VH3-98
	5′GP_VH#19	GAGGTACAGCTCGTGGAATCT	VH3-282
	5′GP_VH#20	GACGTACAGCTCGTGGAGTCT	VH3-265
	5′GP_VH#21	GAGGAGCAACTGGTAGAGTCT	VH3-74
	5′GP_VH#22	GAGCCGCAGCTGGTAGAATCC	VH3-204
	5′GP_VH#23	GCGGAGCAGCTGGTGGAGTCC	VH3-189
	5′GP_VH#24	GAGGTGCAGCTGGTAGAGTCT	VH3-42, VH3-23, VH3-2
	5′GP_VH#25	GAGGTGCAGTTGGTAGAGTCT	VH3-9
	5′GP_VH#26	GATGAGCAACTGGTAGAGTCC	VH3-20
	5′GP_VH#27	GAGGTGCAGCTCATGGAGTCT	VH3-166
	5′GP_VH#28	CAGGTGCAGCTACAGGAGTCA	VH1-68, VH1-59
	5′GP_VH#29	GAGTTGCAGCTGGTGGAGTAC	VH3-222
	5′GP_VH#30	GAGGAGCAGGTGGTGGAGCCC	VH3-36, VH3-8
	5′GP_VH#31	GAGGTACAGCTCGTGGAGTCT	VH3-194
	5′GP_VH#32	CAGGTGAAGCTGCAGGAGTCA	VH2-67
	5′GP_VH#33	GAGCAGCAACTCGTGGAGTCC	VH3-255
	5′GP_VH#34	CAGCTGCAGCTGAAGGAGTCA	VH1-4
	5′GP_VH#35	CAAATGCAGCTGCAAGAGTCA	VH2-145
	5′GP_VH#36	CAGGTGCAGCTGCAGGAGTCC	VH1-215, VH1-209, VH1-84
	5′GP_VH#37	GAGGTGCTGCTGGTGGAGTCT	VH3-48
	5′GP_VH#38	CAGGTGCAGAAGCAGGAGTCA	VH2-70
	5′GP_VH#39	GAGGTGCAGCTGGTGGAGTCC	VH3-114
	5′GP_VH#40	GAGGTACAGCTCGTGGAGGCT	VH3-211
	5′GP_VH#41	CAAATGCAGCTGCAGGAGTCA	VH2-216
	5′GP_VH#42	AAGGTACGGCTGGTGGAGTCC	VH3-190
	5′GP_VH#43	CAGGTGCAGCTGCAAGAGTCG	VH1-140
	5′GP_VH#44	CAGGTTCAGCTGCAAGAGTCA	VH1-151
	5′GP_VH#45	CAGGTGCAGCTGCAGGAGTCA	VH2-239, VH1-171, VH1-156, VH1-101, VH1-77, VH2-160, VH2-94, VH2-86, VH2-54, VH2-43, VH2-35, VH2-28, VH2-27, VH2-17, VH2-7
	5′GP_VH#46	CAGGGGCAGCTGCAGGAGTCA	VH2-121, VH2-109
	5′GP_VH#47	GAGGAGCAGCTGGTGGAGTCC	VH3-229, VH3-186, VH3-185, VH3-183, VH3-51, VH3-24
	5′GP_VH#48	CAGATGCAGCTGCAGGAGTCA	VH1-91
	5′GP_VH#49	GAGGAGCAACTGGTAGAGTCC	VH3-150, VH3-102, VH3-89, VH3-46, VH3-37, VH3-30, VH3-176, VH3-135, VH3-80
	5′GP_VH#50	GAGGCGCAGCTGGTGGAATCC	VH3-197
	5′GP_VH#51	GAGGAGAAGCTGGTGGAGTCT	VH3-137
	5′GP_VH#52	GAGGAGCAGCTGGTGGAGTCT	VH3-287, VH3-271, VH3-104

Reverse primers	3′C_gama_CH1_GP	GGTAGGTGTGCACTCCACTGGTC	Cγ (1st PCR)
	3′IgG(Internal)_GP	GCTCAGGGAAGTAGCCCTTGAC	Cγ (2nd PCR)


**Table 3:** Ig lambda PCR primers.

PCR	Primer	Sequence (5′–3′)	V gene segment
Lamda Chain Forward primers	5′GP_VL#1	CAGCTTGTGCTGACTCAGTCACCCT	VL4-92
	5′GP_VL#2	TCCTATGTGCTGACACAGCCGTCTT	VL3-39
	5′GP_VL#3	TCCTATGTGCTCAAACAGCCACCTT	VL3-30
	5′GP_VL#4	CAGCCGGTGCTCACTCAACCACCCT	VL3-134
	5′GP_VL#5	CAGCCTGTGCTGACTCAGCTTCCCT	VL8-87
	5′GP_VL#6	TCCTATGTACTGACACAGCCATCTT	VL3-27
	5′GP_VL#7	CAGGAACTGGTGACTCAGGAACCCT	VL5-74
	5′GP_VL#8	TCTTACACCCTCACTCAACCTCCCT	VL3-55, VL3-31, VL3-47, VL3-41, VL3-9
	5′GP_VL#9	TCCTATGTATTCACACAGCCACCTT	VL3-6
	5′GP_VL#10	CAGCCTGTGCTGAAACAGTCACCCT	VL4-138, VL4-84, VL4-82
	5′GP_VL#11	CAGGTTGTGCTGACTCAGTCACCCT	VL4-80
	5′GP_VL#12	CAGACTTCAGTCACCCAGGAGCCAT	VL7-129
	5′GP_VL#13	CAGCCTGTGCTGACTCAGCTGCCCT	VL8-104, VL8-85
	5′GP_VL#14	CAGGCAGTGCTGAGTCAGCCGCCCT	VL1-125
	5′GP_VL#15	CAGGATCTGGTGACTCAGGAACCCT	VL5-79, VL5-78
	5′GP_VL#16	CTGGCTGTGGTAACTCAGGAATCTT	VL6-102
	5′GP_VL#17	TCGTATGTGCTGACGCAGCCATCTT	VL3-21
	5′GP_VL#18	TCCTATGTGCTGACACAGCCATCTT	VL3-4
	5′GP_VL#19	CAGTCTGGCCTAAGTCAGGAAGCTT	VL1-96
	5′GP_VL#20	TCCTATGTACTCACACAGCCACCCT	VL3-53
	5′GP_VL#21	AAGGCTGTGGTGACTCAGGAATCTT	VL6-115
	5′GP_VL#22	TCCTATGTACTCACACAGTCACCTT	VL3-24
	5′GP_VL#23	TCCTATGTGCTGACGCAGCCATCTT	VL3-50, VL3-34, VL3-32, VL3-16, VL3-48, VL3-45, VL3-42, VL3-13, VL3-10
	5′GP_VL#24	TCCTATGTACTCACACAGCCACCTT	VL3-52, VL3-29
	5′GP_VL#25	CAGGCTGTGGTGACTCAGGAACCTT	VL6-71
	5′GP_VL#26	CAGCTTGTGCTGACTCAGTCACCTT	VL4-121
	5′GP_VL#27	CAGAATGTAGCGACCCAGGTATCCT	VL5-106
	5′GP_VL#28	CAGACTGTGGTGACCCAGGTATTCT	VL5-122
	5′GP_VL#29	CAGGCAGTGCTGACTCAGCTGCCCT	VL1-95
	5′GP_VL#30	CAGGCAGTGCTGACTCAGCCGCCCT	VL1-109
	5′GP_VL#31	TCTTATATCTTGACACAGCCACCCT	VL3-19
	5′GP_VL#32	TCTTACATCTTGACACAGCCTCCCT	VL3-43_VL3-35
	5′GP_VL#33	CAGGCTGTGGTGACTCAGGAATCTT	VL6-132, VL6-98, VL6-69, VL6-68, VL6-116
	5′GP_VL#34	CAGGATCTGGTAACTCAGGAACCTT	VL5-99
	5′GP_VL#35	TCCTATGTGCTCACACAGCCACCTT	VL3-8
	5′GP_VL#36	CAGCCTGTAGTGACTCAACCACCCT	VL5-139

Reverse primers	3′_C_LM_OUT1_GP	CACCACTGTGGCCTTGTTKTCCTGG	Cλ (1st PCR)
	3′_C_LM_OUT2_GP	CACCACTGTGGCCTTGTTTTCGTTG	Cλ (1st PCR)
	3′XhoI_CL_Int_GP	CTCCTCACTCGAGGGYGGGAAYAGGCTG	Cλ (2nd PCR)


**Table 4:** Ig kappa PCR primers.

PCR	Primer	Sequence (5′–3′)	V gene segment
**IGK PCR primers**			
Kappa Chain Forward primers	5′GP_VK#1	GACATCCAGATGACCCAGTCTCCAT	Vk1-142
	5′GP_VK#2	GACATTGTTATGACCCAGTCTACAG	Vk4-36
	5′GP_VK#3	GCATCCAGTTGACACAGCCTCCATC	Vk1-178
	5′GP_VK#4	GTCATCCAGATGATGCAGTATTCAT	Vk3-108
	5′GP_VK#5	GATATCCAGTTGACACAGCCTGCAT	Vk1-147, Vk1-58
	5′GP_VK#6	GACATTTTGATGACCCAGTCTCCAG	Vk4-37
	5′GP_VK#7	GAAATTCAGATGACACAAACTTCCT	Vk1-99
	5′GP_VK#8	GAAGTTGTGCTGACCCAGACTCCAC	Vk2-124
	5′GP_VK#9	GACATCCAGATGATCCAGTCACCAG	Vk1-133
	5′GP_VK#10	GACATCCAGATTACTCAGACTCCAT	Vk1-174
	5′GP_VK#11	GACATCCAGATGACTCAGACTCCAT	Vk1-280, Vk1-226, Vk1-55, Vk1-51, Vk1-47, Vk1-40, Vk1-15
	5′GP_VK#12	GACATCCAGATGACTCAGACTCCGT	Vk1-272
	5′GP_VK#13	GAAACCCTGCTGACTGAGACTCCAG	Vk3-25
	5′GP_VK#14	GACATCCAGTTGACGCAGCCTCCAT	Vk1-201
	5′GP_VK#15	GATGTTCTGATGACCCAGACCCCAC	Vk2-15
	5′GP_VK#16	GATGTAGTGATGACCCAGACTCCAC	Vk2-16
	5′GP_VK#17	GATACTCAGATGACTCAGTCTCCAT	Vk1-247
	5′GP_VK#18	GAAACCCTTCTGACACAGACCCCAG	Vk3-155
	5′GP_VK#19	GATGTTGTGGTGACCCAGACCCCAC	Vk2-51, Vk2-11
	5′GP_VK#20	GATATCCAGATGACTCAGGCTCCTT	Vk1-230
	5′GP_VK#21	GACATCCAGATGATTCAGACTCCAT	Vk1-21
	5′GP_VK#22	GACATTGTGATGACTCAGTCTCCAG	Vk4-28
	5′GP_VK#23	GACATCCAGTTAACACAGCCTCCAT	Vk1-211, Vk1-159
	5′GP_VK#24	GACATCCAGTTGACCCAGTCTCCAT	Vk1-246
	5′GP_VK#25	GACATTAGGATGACCCAGACCCCAC	Vk2-85
	5′GP_VK#26	GATGTTGTATTGACCCAAACCCCAC	Vk2-14
	5′GP_VK#27	GACATCCAGATGACCCAGTCACCAT	Vk1-190
	5′GP_VK#28	GACATTGTGATGACCCAGTCTCCAG	Vk4-70, Vk4-95, Vk4-45, Vk4-41, Vk4-39, Vk4-32, Vk4-29, Vk4-90, Vk4-83, Vk4-81
	5′GP_VK#29	GAAACCCTGTTGACCCAGACTCCAG	Vk3-170
	5′GP_VK#30	GATATCCAGTTGACACAGCCTCCAT	Vk1-152
	5′GP_VK#31	GACATTGTGATGACCCAGTCACCAG	Vk4-74
	5′GP_VK#32	GAAATTGTGATGACCCAGTCTCCAG	Vk4-77
	5′GP_VK#33	GACATCCCGATGACTCAGATTCCAT	Vk1-36
	5′GP_VK#34	GACATTCAGATGACCCAGTCTCCAT	Vk1-140, Vk1-65, Vk1-254
	5′GP_VK#35	GACATACAGATGACCCAGTGTCCAT	Vk1-234
	5′GP_VK#36	GACAATGTGGTGATCCAGTCTCCAG	Vk4-69
	5′GP_VK#37	GACATCCAGTTGACACAGCCTCCTT	Vk1-214
	5′GP_VK#38	GACATCCAGATGACCCAGTCTCAAT	Vk1-144
	5′GP_VK#39	GACACCCAGATGACCCAGTCTCCAT	Vk1-145
	5′GP_VK#40	GACATCCAGATGACTCAGACTGCAT	Vk1-59
	5′GP_VK#41	GACTTCCAGATGACCCAGTCACCAT	Vk1-219
	5′GP_VK#42	GACATCCAGTTGACACAGCCTCCAT	Vk1-242, Vk1-239
	5′GP_VK#43	GACATAGTGATGACCCAGACCCCAC	Vk2-10
	5′GP_VK#44	GATGTTGTGATGACCCAGACCGCAC	Vk2-7
	5′GP_VK#45	GACATCCGGATGACTCAGACTCCAT	Vk1-179
	5′GP_VK#46	GAAAAATTACTGACTAAGACTCCAG	Vk3-263
	5′GP_VK#47	GATATCCAGATGACTCAGGCTCCCT	Vk1-267
	5′GP_VK#48	GACATCCAATTGACACAGCCTGCAT	Vk1-279, Vk1-266, Vk1-29, Vk1-18, Vk1-1
	5′GP_VK#49	GAAACCCAGCTGACTCAGACTCCAG	Vk3-281, Vk3-60, Vk3-42
	5′GP_VK#50	GATATTGTGATGACACAGACCCCAC	Vk2-20
	5′GP_VK#51	GACATCCAGTTGACCCAGACTCCAG	Vk1-126
	5′GP_VK#52	GACATCCTATTAACCCAGCCTCCCT	Vk1-101
	5′GP_VK#53	CAAATTGTGCTCACCCAGACTCCAG	Vk5-57
	5′GP_VK#54	GATGTTTTGATGACCCAGACCCCAC	Vk2-22
	5′GP_VK#55	GAAATTGTGCTTACCCAGTCTCCAG	Vk5-105
	5′GP_VK#56	GATATCCAGTTGACCCAGTCTTCCT	Vk1-117
	5′GP_VK#57	GACATCAAATTGACTCAGCCAGCAT	Vk1-233
	5′GP_VK#58	GTGTAGGAAAAAACATCACTATTAC	Vk1-245
	5′GP_VK#59	GACATCCAGATGACTCAGACTCTCT	Vk1-153
	5′GP_VK#60	GACATCCAGTTGACCCAGTCTCCCT	Vk1-131, Vk1-115, Vk1-65
	5′GP_VK#61	GACCTTGTTATGACACAGTCTCCAG	Vk4-76
	5′GP_VK#62	GACATTCAGATGAGCCAGTCTCCAT	Vk1-184
	5′GP_VK#63	GACATCCAGTTGATGCAGCCTCCAT	Vk1-253
	5′GP_VK#64	GATGTTGTGATGACCCAGACCCCAC	Vk2-3
	5′GP_VK#65	GACATCCAACTGACACAACCTGCAT	Vk1-261, Vk1-203
	5′GP_VK#66	GACATCCAGATGACTCAGTCTCCCT	Vk1-130, Vk1-128
	5′GP_VK#67	GACATCCAAATGACTCAGGTTCCAT	Vk1-166
	5′GP_VK#68	GATGTTTTGTTGTCCCAGACCCCAC	Vk2-2
	5′GP_VK#69	GACATCCAGTTGACACAGCCTGCAT	Vk1-275, Vk1-271, Vk1-225, Vk1-54, Vk1-50, Vk1-46, Vk1-45, Vk1-39, Vk1-35, Vk1-24, Vk1-20, Vk1-14, Vk1-8
	5′GP_VK#70	GACATCCAGCTGACACAGCTTGCAT	Vk1-11
	5′GP_VK#71	GCGTTGCCCTGACACAGTCCCCAGC	Vk6-164

Reverse primers	3′ Ck 156_OUT_GP	GTGTTGTCCTTGCTGTCCTGATC	Ck (1st PCR)
	3′Ck_internal_GP	GTTCAGAGCCATCCACCTTCCAC	Ck (2nd PCR)


### Single B Cell Ig Gene Sequence Analysis

Sequences of the semi-nested PCR products were initially analyzed by IMGT/High V-Quest (Alamyar et al., 2012) to define Ig gene structure, particularly the framework and CDR boundaries, using human Ig sequences as reference. The V(D)J sequences identified were further annotated by the stand-alone software IgBLAST (Ye et al., 2013) using previously annotated guinea pig germline sequences (Guo et al., 2012) as reference, which were annotated from guinea pig genome database^1^. Somatic hypermutation (SHM) level (Mut %) was calculated as the divergence of antibody VH/VL/VK sequences from the assigned germline sequences at nucleotide level. Clonal lineages were defined by the usage of V and J segments, and CDR3 homology (>80% homology).

### Cloning and Expression of Guinea Pig Monoclonal Antibodies in a Guinea Pig-Human Chimeric Form

After sequence annotation, the VH/VK/VL amplicons from single cell RT-PCR were inserted into human IgG1 expression vectors (Tiller et al., 2008) by seamless cloning as described next to form guinea pig-human chimeric mAbs. Amplicons were subjected to another round of PCR amplification (cloning PCR) using seamless cloning primers, which contain VH/VK/VL gene-specific regions and additional overhangs identical to the sequences in the expression vectors (Figure 2C). The products of cloning PCR reactions were purified and inserted into expression vectors by seamless cloning. Seamless cloning primers designed for VH, VK, and VL amplification and cloning are summarized in Figure 2C and Tables 5–7. The primers for each cloning PCR were selected based on germline V and J gene segment usage derived from the Ig gene sequence analysis. The cloning PCR reaction was performed in a total volume of 50 μl with high-fidelity DNA polymerase (Roche). The PCR reaction mixture consisted of 1 μl of template using the 2nd PCR product from the single cell RT-PCR reaction, 5 μl of 10× reaction buffer, 1 μl of 10 mM dNTPs, 1 μl of 25 μM of 5′ and 3′ cloning primers, 1 μl of high-fidelity DNA polymerase (3.5 Unit/μl, Roche) and nuclease-free water. The PCR program had an initial denaturation at 95°C for 3 min, followed by 20 cycles of 95°C for 30 s, 50°C for 30 s, and 68°C for 2 min. There was a final elongation step at 68°C for 8 min. The products were evaluated on 1% agarose gels before being assembled into their respective expression vectors containing human Igγ1H, Igκ1L, or Igλ2L constant regions described previously (Tiller et al., 2008). The assembly (insertion) reactions were performed with GeneArt assembly enzyme mix (Invitrogen) per manufacturer’s instructions.

**Table 5:** Ig heavy cloning primers.

Cloning PCR	Primer	Sequence (5′–3′)	V gene segment
**IGH PCR primers**			
Heavy Chain Forward primers	SL_5′GP_VH#1	tttctagtagcaactgcaaccggtgtacattctGAGGAGCAACTGGTGGAGTCC	VH3-157, VH3-139
	SL_5′GP_VH#2	tttctagtagcaactgcaaccggtgtacattctCAGTTGCAGCTGCAGGAGTCA	VH1-14
	SL_5′GP_VH#3	tttctagtagcaactgcaaccggtgtacattctCAGGTGCAGCTGCAGGAGTCG	VH1-120, VH1-115, VH1-58
	SL_5′GP_VH#4	tttctagtagcaactgcaaccggtgtacattctCAGGTGCAGCTGCAGGAGTTG	VH1-56
	SL_5′GP_VH#5	tttctagtagcaactgcaaccggtgtacattctGAAGTACAGCTCACACAATCT	VH3-184
	SL_5′GP_VH#6	tttctagtagcaactgcaaccggtgtacattctGAGGTGCAGCTCGTGGAGTCT	VH3-170, VH3-167
	SL_5′GP_VH#7	tttctagtagcaactgcaaccggtgtacattctCAGGTTCAGCTGCAGGAGTCG	VH1-66, VH1-75
	SL_5′GP_VH#8	tttctagtagcaactgcaaccggtgtacattctCAGGTTCAGCTGCAGGAGTCA	VH1-1
	SL_5′GP_VH#9	tttctagtagcaactgcaaccggtgtacattctGAGGAGCAGCTGGTAGAGTCC	VH3-63, VH3-3
	SL_5′GP_VH#10	tttctagtagcaactgcaaccggtgtacattctGAGGTGCAGCTGGTGGAGTCT	VH3-35
	SL_5′GP_VH#11	tttctagtagcaactgcaaccggtgtacattctGAGGTGCAGCTGATGGAGTCC	VH3-230
	SL_5′GP_VH#12	tttctagtagcaactgcaaccggtgtacattctCAGGTGCAGCTACAGGAGTCG	VH1-95
	SL_5′GP_VH#13	tttctagtagcaactgcaaccggtgtacattctGAAGAGCAGCTGGTGGAGTCT	VH3-80
	SL_5′GP_VH#14	tttctagtagcaactgcaaccggtgtacattctGAGCCGCAGCTGGTGGAGTCA	VH3-194
	SL_5′GP_VH#15	tttctagtagcaactgcaaccggtgtacattctGAAGTACAGCTCACACAGTCT	VH3-201
	SL_5′GP_VH#16	tttctagtagcaactgcaaccggtgtacattctGAAGTGCAGCTCGTGGAGTCC	VH3-262
	SL_5′GP_VH#17	tttctagtagcaactgcaaccggtgtacattctGAAGTGCAACTCGTGGAGTCC	VH3-126
	SL_5′GP_VH#18	tttctagtagcaactgcaaccggtgtacattctGGTGCAGCTGGTGGAGTCCGG	VH3-98
	SL_5′GP_VH#19	tttctagtagcaactgcaaccggtgtacattctGAGGTACAGCTCGTGGAATCT	VH3-282
	SL_5′GP_VH#20	tttctagtagcaactgcaaccggtgtacattctGACGTACAGCTCGTGGAGTCT	VH3-265
	SL_5′GP_VH#21	tttctagtagcaactgcaaccggtgtacattctGAGGAGCAACTGGTAGAGTCT	VH3-74
	SL_5′GP_VH#22	tttctagtagcaactgcaaccggtgtacattctGAGCCGCAGCTGGTAGAATCC	VH3-204
	SL_5′GP_VH#23	tttctagtagcaactgcaaccggtgtacattctGCGGAGCAGCTGGTGGAGTCC	VH3-189
	SL_5′GP_VH#24	tttctagtagcaactgcaaccggtgtacattctGAGGTGCAGCTGGTAGAGTCT	VH3-42, VH3-23, VH3-2
	SL_5′GP_VH#25	tttctagtagcaactgcaaccggtgtacattctGAGGTGCAGTTGGTAGAGTCT	VH3-9
	SL_5′GP_VH#26	tttctagtagcaactgcaaccggtgtacattctGATGAGCAACTGGTAGAGTCC	VH3-20
	SL_5′GP_VH#27	tttctagtagcaactgcaaccggtgtacattctGAGGTGCAGCTCATGGAGTCT	VH3-166
	SL_5′GP_VH#28	tttctagtagcaactgcaaccggtgtacattctCAGGTGCAGCTACAGGAGTCA	VH1-68, VH1-59
	SL_5′GP_VH#29	tttctagtagcaactgcaaccggtgtacattctGAGTTGCAGCTGGTGGAGTAC	VH3-222
	SL_5′GP_VH#30	tttctagtagcaactgcaaccggtgtacattctGAGGAGCAGGTGGTGGAGCCC	VH3-36, VH3-8
	SL_5′GP_VH#31	tttctagtagcaactgcaaccggtgtacattctGAGGTACAGCTCGTGGAGTCT	VH3-194
	SL_5′GP_VH#32	tttctagtagcaactgcaaccggtgtacattctCAGGTGAAGCTGCAGGAGTCA	VH2-67
	SL_5′GP_VH#33	tttctagtagcaactgcaaccggtgtacattctGAGCAGCAACTCGTGGAGTCC	VH3-255
	SL_5′GP_VH#34	tttctagtagcaactgcaaccggtgtacattctCAGCTGCAGCTGAAGGAGTCA	VH1-4
	SL_5′GP_VH#35	tttctagtagcaactgcaaccggtgtacattctCAAATGCAGCTGCAAGAGTCA	VH2-145
	SL_5′GP_VH#36	tttctagtagcaactgcaaccggtgtacattctCAGGTGCAGCTGCAGGAGTCC	VH1-215, VH1-209, VH1-84
	SL_5′GP_VH#37	tttctagtagcaactgcaaccggtgtacattctGAGGTGCTGCTGGTGGAGTCT	VH3-48
	SL_5′GP_VH#38	tttctagtagcaactgcaaccggtgtacattctCAGGTGCAGAAGCAGGAGTCA	VH2-70
	SL_5′GP_VH#39	tttctagtagcaactgcaaccggtgtacattctGAGGTGCAGCTGGTGGAGTCC	VH3-114
	SL_5′GP_VH#40	tttctagtagcaactgcaaccggtgtacattctGAGGTACAGCTCGTGGAGGCT	VH3-211
	SL_5′GP_VH#41	tttctagtagcaactgcaaccggtgtacattctCAAATGCAGCTGCAGGAGTCA	VH2-216
	SL_5′GP_VH#42	tttctagtagcaactgcaaccggtgtacattctAAGGTACGGCTGGTGGAGTCC	VH3-190
	SL_5′GP_VH#43	tttctagtagcaactgcaaccggtgtacattctCAGGTGCAGCTGCAAGAGTCG	VH1-140
	SL_5′GP_VH#44	tttctagtagcaactgcaaccggtgtacattctCAGGTTCAGCTGCAAGAGTCA	VH1-151
	SL_5′GP_VH#45	tttctagtagcaactgcaaccggtgtacattctCAGGTGCAGCTGCAGGAGTCA	VH2-239, VH1-171, VH1-156, VH1-101, VH1-77, VH2-160, VH2-94, VH2-86, VH2-54, VH2-43, VH2-35, VH2-28, VH2-27, VH2-17, VH2-7
	SL_5′GP_VH#46	tttctagtagcaactgcaaccggtgtacattctCAGGGGCAGCTGCAGGAGTCA	VH2-121, VH2-109
	SL_5′GP_VH#47	tttctagtagcaactgcaaccggtgtacattctGAGGAGCAGCTGGTGGAGTCC	VH3-229, VH3-186, VH3-185, VH3-183, VH3-51, VH3-24
	SL_5′GP_VH#48	tttctagtagcaactgcaaccggtgtacattctCAGATGCAGCTGCAGGAGTCA	VH1-91
	SL_5′GP_VH#49	tttctagtagcaactgcaaccggtgtacattctGAGGAGCAACTGGTAGAGTCC	VH3-150, VH3-102, VH3-89, VH3-46, VH3-37, VH3-30, VH3-176, VH3-135, VH3-80
	SL_5′GP_VH#50	tttctagtagcaactgcaaccggtgtacattctGAGGCGCAGCTGGTGGAATCC	VH3-197
	SL_5′GP_VH#51	tttctagtagcaactgcaaccggtgtacattctGAGGAGAAGCTGGTGGAGTCT	VH3-137
	SL_5′GP_VH#52	tttctagtagcaactgcaaccggtgtacattctGAGGAGCAGCTGGTGGAGTCT	VH3-287, VH3-271, VH3-104

Reverse primers	3′ *Sal*I JH 1_GP	agaccgatgggcccttggtcgacGCTGACGTGACGGTGACTGAG	JH 1
	3′ *Sal*I JH 2_4_6_GP	agaccgatgggcccttggtcgacGCTGAGGAGACGGTGACCAG	JH 2, JH 4, JH6
	3′ *Sal*I JH 3_GP	agaccgatgggcccttggtcgacGCTGAGGAGATAGTGACCAG	JH 3
	3′ *Sal*I JH 5_GP	agaccgatgggcccttggtcgacGCTGAGGAGACGGTGACCGA	JH 5


**Table 6:** Ig lambda cloning primers.

Cloning PCR	Primer	Sequence (5′–3′)	V gene segment
**IGL PCR primers**			
Lamda Chain Forward primers	SL_5′GP_VL#1	tttctagtagcaactgcaaccggttctctctcgCAGCTTGTGCTGACTCAGTCACCCT	VL4-92
	SL_5′GP_VL#2	tttctagtagcaactgcaaccggttctctctcgTCCTATGTGCTGACACAGCCGTCTT	VL3-39
	SL_5′GP_VL#3	tttctagtagcaactgcaaccggttctctctcgTCCTATGTGCTCAAACAGCCACCTT	VL3-30
	SL_5′GP_VL#4	tttctagtagcaactgcaaccggttctctctcgCAGCCGGTGCTCACTCAACCACCCT	VL3-134
	SL_5′GP_VL#5	tttctagtagcaactgcaaccggttctctctcgCAGCCTGTGCTGACTCAGCTTCCCT	VL8-87
	SL_5′GP_VL#6	tttctagtagcaactgcaaccggttctctctcgTCCTATGTACTGACACAGCCATCTT	VL3-27
	SL_5′GP_VL#7	tttctagtagcaactgcaaccggttctctctcgCAGGAACTGGTGACTCAGGAACCCT	VL5-74
	SL_5′GP_VL#8	tttctagtagcaactgcaaccggttctctctcgTCTTACACCCTCACTCAACCTCCCT	VL3-55, VL3-31, VL3-47, VL3-41, VL3-9
	SL_5′GP_VL#9	tttctagtagcaactgcaaccggttctctctcgTCCTATGTATTCACACAGCCACCTT	VL3-6
	SL_5′GP_VL#10	tttctagtagcaactgcaaccggttctctctcgCAGCCTGTGCTGAAACAGTCACCCT	VL4-138, VL4-84, VL4-82
	SL_5′GP_VL#11	tttctagtagcaactgcaaccggttctctctcgCAGGTTGTGCTGACTCAGTCACCCT	VL4-80
	SL_5′GP_VL#12	tttctagtagcaactgcaaccggttctctctcgCAGACTTCAGTCACCCAGGAGCCAT	VL7-129
	SL_5′GP_VL#13	tttctagtagcaactgcaaccggttctctctcgCAGCCTGTGCTGACTCAGCTGCCCT	VL8-104, VL8-85
	SL_5′GP_VL#14	tttctagtagcaactgcaaccggttctctctcgCAGGCAGTGCTGAGTCAGCCGCCCT	VL1-125
	SL_5′GP_VL#15	tttctagtagcaactgcaaccggttctctctcgCAGGATCTGGTGACTCAGGAACCCT	VL5-79, VL5-78
	SL_5′GP_VL#16	tttctagtagcaactgcaaccggttctctctcgCTGGCTGTGGTAACTCAGGAATCTT	VL6-102
	SL_5′GP_VL#17	tttctagtagcaactgcaaccggttctctctcgTCGTATGTGCTGACGCAGCCATCTT	VL3-21
	SL_5′GP_VL#18	tttctagtagcaactgcaaccggttctctctcgTCCTATGTGCTGACACAGCCATCTT	VL3-4
	SL_5′GP_VL#19	tttctagtagcaactgcaaccggttctctctcgCAGTCTGGCCTAAGTCAGGAAGCTT	VL1-96
	SL_5′GP_VL#20	tttctagtagcaactgcaaccggttctctctcgTCCTATGTACTCACACAGCCACCCT	VL3-53
	SL_5′GP_VL#21	tttctagtagcaactgcaaccggttctctctcgAAGGCTGTGGTGACTCAGGAATCTT	VL6-115
	SL_5′GP_VL#22	tttctagtagcaactgcaaccggttctctctcgTCCTATGTACTCACACAGTCACCTT	VL3-24
	SL_5′GP_VL#23	tttctagtagcaactgcaaccggttctctctcgTCCTATGTGCTGACGCAGCCATCTT	VL3-50, VL3-34, VL3-32, VL3-16, VL3-48, VL3-45, VL3-42, VL3-13, VL3-10
	SL_5′GP_VL#24	tttctagtagcaactgcaaccggttctctctcgTCCTATGTACTCACACAGCCACCTT	VL3-52, VL3-29
	SL_5′GP_VL#25	tttctagtagcaactgcaaccggttctctctcgCAGGCTGTGGTGACTCAGGAACCTT	VL6-71
	SL_5′GP_VL#26	tttctagtagcaactgcaaccggttctctctcgCAGCTTGTGCTGACTCAGTCACCTT	VL4-121
	SL_5′GP_VL#27	tttctagtagcaactgcaaccggttctctctcgCAGAATGTAGCGACCCAGGTATCCT	VL5-106
	SL_5′GP_VL#28	tttctagtagcaactgcaaccggttctctctcgCAGACTGTGGTGACCCAGGTATTCT	VL5-122
	SL_5′GP_VL#29	tttctagtagcaactgcaaccggttctctctcgCAGGCAGTGCTGACTCAGCTGCCCT	VL1-95
	SL_5′GP_VL#30	tttctagtagcaactgcaaccggttctctctcgCAGGCAGTGCTGACTCAGCCGCCCT	VL1-109
	SL_5′GP_VL#31	tttctagtagcaactgcaaccggttctctctcgTCTTATATCTTGACACAGCCACCCT	VL3-19
	SL_5′GP_VL#32	tttctagtagcaactgcaaccggttctctctcgTCTTACATCTTGACACAGCCTCCCT	VL3-43_VL3-35
	SL_5′GP_VL#33	tttctagtagcaactgcaaccggttctctctcgCAGGCTGTGGTGACTCAGGAATCTT	VL6-132, VL6-98, VL6-69, VL6-68, VL6-116
	SL_5′GP_VL#34	tttctagtagcaactgcaaccggttctctctcgCAGGATCTGGTAACTCAGGAACCTT	VL5-99
	SL_5′GP_VL#35	tttctagtagcaactgcaaccggttctctctcgTCCTATGTGCTCACACAGCCACCTT	VL3-8
	SL_5′GP_VL#36	tttctagtagcaactgcaaccggttctctctcgCAGCCTGTAGTGACTCAACCACCCT	VL5-139
Reverse primers	3_GP_*Xho*I_JL_1_4_7	ggcttgaagctcctcactcgagggcgggaacagagtgaccaagggggaagccttgggctgaccg AGGACGGTCAGCTTGGTG	JL1, JL4, JL7
	3_GP_*Xho*I_JL_2_5_9	ggcttgaagctcctcactcgagggcgggaacagagtgaccaagggggaagccttgggctgaccg AGGACCGTCAGCCTGGTT	JL2, JL5, JL6
	3_GP_*Xho*I_JL3_6_10	ggcttgaagctcctcactcgagggcgggaacagagtgaccaagggggaagccttgggctgaccg AGGACGGTCAGCTTGGTT	JL3, JL6, JL10
	3_GP_*Xho*I_JL8	ggcttgaagctcctcactcgagggcgggaacagagtgaccaagggggaagccttgggctgaccg AGGACTGTCAGGTCGGTT	JL8
	3_GP_*Xho*I_JL11	ggcttgaagctcctcactcgagggcgggaacagagtgaccaagggggaagccttgggctgaccg AGGACGGTCACCTTGGTC	JL11


**Table 7:** Ig kappa cloning primers.

Cloning PCR	Primer	Sequence (5′–3′)	V gene segment
**IGK PCR primers**			
Kappa Chain Forward primers	SL_5′GP_VK#1	tttctagtagcaactgcaaccggtgtacattctGACATCCAGATGACCCAGTCTCCAT	Vk1-142
	SL_5′GP_VK#2	tttctagtagcaactgcaaccggtgtacattctGACATTGTTATGACCCAGTCTACAG	Vk4-36
	SL_5′GP_VK#3	tttctagtagcaactgcaaccggtgtacattctGCATCCAGTTGACACAGCCTCCATC	Vk1-178
	SL_5′GP_VK#4	tttctagtagcaactgcaaccggtgtacattctGTCATCCAGATGATGCAGTATTCAT	Vk3-108
	SL_5′GP_VK#5	tttctagtagcaactgcaaccggtgtacattctGATATCCAGTTGACACAGCCTGCAT	Vk1-147, Vk1-58
	SL_5′GP_VK#6	tttctagtagcaactgcaaccggtgtacattctGACATTTTGATGACCCAGTCTCCAG	Vk4-37
	SL_5′GP_VK#7	tttctagtagcaactgcaaccggtgtacattctGAAATTCAGATGACACAAACTTCCT	Vk1-99
	SL_5′GP_VK#8	tttctagtagcaactgcaaccggtgtacattctGAAGTTGTGCTGACCCAGACTCCAC	Vk2-124
	SL_5′GP_VK#9	tttctagtagcaactgcaaccggtgtacattctGACATCCAGATGATCCAGTCACCAG	Vk1-133
	SL_5′GP_VK#10	tttctagtagcaactgcaaccggtgtacattctGACATCCAGATTACTCAGACTCCAT	Vk1-174
	SL_5′GP_VK#11	tttctagtagcaactgcaaccggtgtacattctGACATCCAGATGACTCAGACTCCAT	Vk1-280, Vk1-226, Vk1-55, Vk1-51, Vk1-47, Vk1-40, Vk1-15
	SL_5′GP_VK#12	tttctagtagcaactgcaaccggtgtacattctGACATCCAGATGACTCAGACTCCGT	Vk1-272
	SL_5′GP_VK#13	tttctagtagcaactgcaaccggtgtacattctGAAACCCTGCTGACTGAGACTCCAG	Vk3-25
	SL_5′GP_VK#14	tttctagtagcaactgcaaccggtgtacattctGACATCCAGTTGACGCAGCCTCCAT	Vk1-201
	SL_5′GP_VK#15	tttctagtagcaactgcaaccggtgtacattctGATGTTCTGATGACCCAGACCCCAC	Vk2-15
	SL_5′GP_VK#16	tttctagtagcaactgcaaccggtgtacattctGATGTAGTGATGACCCAGACTCCAC	Vk2-16
	SL_5′GP_VK#17	tttctagtagcaactgcaaccggtgtacattctGATACTCAGATGACTCAGTCTCCAT	Vk1-247
	SL_5′GP_VK#18	tttctagtagcaactgcaaccggtgtacattctGAAACCCTTCTGACACAGACCCCAG	Vk3-155
	SL_5′GP_VK#19	tttctagtagcaactgcaaccggtgtacattctGATGTTGTGGTGACCCAGACCCCAC	Vk2-51, Vk2-11
	SL_5′GP_VK#20	tttctagtagcaactgcaaccggtgtacattctGATATCCAGATGACTCAGGCTCCTT	Vk1-230
	SL_5′GP_VK#21	tttctagtagcaactgcaaccggtgtacattctGACATCCAGATGATTCAGACTCCAT	Vk1-21
	SL_5′GP_VK#22	tttctagtagcaactgcaaccggtgtacattctGACATTGTGATGACTCAGTCTCCAG	Vk4-28
	SL_5′GP_VK#23	tttctagtagcaactgcaaccggtgtacattctGACATCCAGTTAACACAGCCTCCAT	Vk1-211, Vk1-159
	SL_5′GP_VK#24	tttctagtagcaactgcaaccggtgtacattctGACATCCAGTTGACCCAGTCTCCAT	Vk1-246
	SL_5′GP_VK#25	tttctagtagcaactgcaaccggtgtacattctGACATTAGGATGACCCAGACCCCAC	Vk2-85
	SL_5′GP_VK#26	tttctagtagcaactgcaaccggtgtacattctGATGTTGTATTGACCCAAACCCCAC	Vk2-14
	SL_5′GP_VK#27	tttctagtagcaactgcaaccggtgtacattctGACATCCAGATGACCCAGTCACCAT	Vk1-190
	SL_5′GP_VK#28	tttctagtagcaactgcaaccggtgtacattctGACATTGTGATGACCCAGTCTCCAG	Vk4-70, Vk4-95, Vk4-45, Vk4-41, Vk4-39, Vk4-32, Vk4-29, Vk4-90, Vk4-83, Vk4-81
	SL_5′GP_VK#29	tttctagtagcaactgcaaccggtgtacattctGAAACCCTGTTGACCCAGACTCCAG	Vk3-170
	SL_5′GP_VK#30	tttctagtagcaactgcaaccggtgtacattctGATATCCAGTTGACACAGCCTCCAT	Vk1-152
	SL_5′GP_VK#31	tttctagtagcaactgcaaccggtgtacattctGACATTGTGATGACCCAGTCACCAG	Vk4-74
	SL_5′GP_VK#32	tttctagtagcaactgcaaccggtgtacattctGAAATTGTGATGACCCAGTCTCCAG	Vk4-77
	SL_5′GP_VK#33	tttctagtagcaactgcaaccggtgtacattctGACATCCCGATGACTCAGATTCCAT	Vk1-36
	SL_5′GP_VK#34	tttctagtagcaactgcaaccggtgtacattctGACATTCAGATGACCCAGTCTCCAT	Vk1-140, Vk1-65, Vk1-254
	SL_5′GP_VK#35	tttctagtagcaactgcaaccggtgtacattctGACATACAGATGACCCAGTGTCCAT	Vk1-234
	SL_5′GP_VK#36	tttctagtagcaactgcaaccggtgtacattctGACAATGTGGTGATCCAGTCTCCAG	Vk4-69
	SL_5′GP_VK#37	tttctagtagcaactgcaaccggtgtacattctGACATCCAGTTGACACAGCCTCCTT	Vk1-214
	SL_5′GP_VK#38	tttctagtagcaactgcaaccggtgtacattctGACATCCAGATGACCCAGTCTCAAT	Vk1-144
	SL_5′GP_VK#39	tttctagtagcaactgcaaccggtgtacattctGACACCCAGATGACCCAGTCTCCAT	Vk1-145
	SL_5′GP_VK#40	tttctagtagcaactgcaaccggtgtacattctGACATCCAGATGACTCAGACTGCAT	Vk1-59
	SL_5′GP_VK#41	tttctagtagcaactgcaaccggtgtacattctGACTTCCAGATGACCCAGTCACCAT	Vk1-219
	SL_5′GP_VK#42	tttctagtagcaactgcaaccggtgtacattctGACATCCAGTTGACACAGCCTCCAT	Vk1-242, Vk1-239
	SL_5′GP_VK#43	tttctagtagcaactgcaaccggtgtacattctGACATAGTGATGACCCAGACCCCAC	Vk2-10
	SL_5′GP_VK#44	tttctagtagcaactgcaaccggtgtacattctGATGTTGTGATGACCCAGACCGCAC	Vk2-7
	SL_5′GP_VK#45	tttctagtagcaactgcaaccggtgtacattctGACATCCGGATGACTCAGACTCCAT	Vk1-179
	SL_5′GP_VK#46	tttctagtagcaactgcaaccggtgtacattctGAAAAATTACTGACTAAGACTCCAG	Vk3-263
	SL_5′GP_VK#47	tttctagtagcaactgcaaccggtgtacattctGATATCCAGATGACTCAGGCTCCCT	Vk1-267
	SL_5′GP_VK#48	tttctagtagcaactgcaaccggtgtacattctGACATCCAATTGACACAGCCTGCAT	Vk1-279, Vk1-266, Vk1-29, Vk1-18, Vk1-1
	SL_5′GP_VK#49	tttctagtagcaactgcaaccggtgtacattctGAAACCCAGCTGACTCAGACTCCAG	Vk3-281_Vk3-60_Vk3-42
	SL_5′GP_VK#50	tttctagtagcaactgcaaccggtgtacattctGATATTGTGATGACACAGACCCCAC	Vk2-20
	SL_5′GP_VK#51	tttctagtagcaactgcaaccggtgtacattctGACATCCAGTTGACCCAGACTCCAG	Vk1-126
	SL_5′GP_VK#52	tttctagtagcaactgcaaccggtgtacattctGACATCCTATTAACCCAGCCTCCCT	Vk1-101
	SL_5′GP_VK#53	tttctagtagcaactgcaaccggtgtacattctCAAATTGTGCTCACCCAGACTCCAG	Vk5-57
	SL_5′GP_VK#54	tttctagtagcaactgcaaccggtgtacattctGATGTTTTGATGACCCAGACCCCAC	Vk2-22
	SL_5′GP_VK#55	tttctagtagcaactgcaaccggtgtacattctGAAATTGTGCTTACCCAGTCTCCAG	Vk5-105
	SL_5′GP_VK#56	tttctagtagcaactgcaaccggtgtacattctGATATCCAGTTGACCCAGTCTTCCT	Vk1-117
	SL_5′GP_VK#57	tttctagtagcaactgcaaccggtgtacattctGACATCAAATTGACTCAGCCAGCAT	Vk1-233
	SL_5′GP_VK#58	tttctagtagcaactgcaaccggtgtacattctGTGTAGGAAAAAACATCACTATTAC	Vk1-245
	SL_5′GP_VK#59	tttctagtagcaactgcaaccggtgtacattctGACATCCAGATGACTCAGACTCTCT	Vk1-153
	SL_5′GP_VK#60	tttctagtagcaactgcaaccggtgtacattctGACATCCAGTTGACCCAGTCTCCCT	Vk1-131, Vk1-115, Vk1-65
	SL_5′GP_VK#61	tttctagtagcaactgcaaccggtgtacattctGACCTTGTTATGACACAGTCTCCAG	Vk4-76
	SL_5′GP_VK#62	tttctagtagcaactgcaaccggtgtacattctGACATTCAGATGAGCCAGTCTCCAT	Vk1-184
	SL_5′GP_VK#63	tttctagtagcaactgcaaccggtgtacattctGACATCCAGTTGATGCAGCCTCCAT	Vk1-253
	SL_5′GP_VK#64	tttctagtagcaactgcaaccggtgtacattctGATGTTGTGATGACCCAGACCCCAC	Vk2-3
	SL_5′GP_VK#65	tttctagtagcaactgcaaccggtgtacattctGACATCCAACTGACACAACCTGCAT	Vk1-261, Vk1-203
	SL_5′GP_VK#66	tttctagtagcaactgcaaccggtgtacattctGACATCCAGATGACTCAGTCTCCCT	Vk1-130, Vk1-128
	SL_5′GP_VK#67	tttctagtagcaactgcaaccggtgtacattctGACATCCAAATGACTCAGGTTCCAT	Vk1-166
	SL_5′GP_VK#68	tttctagtagcaactgcaaccggtgtacattctGATGTTTTGTTGTCCCAGACCCCAC	Vk2-2
	SL_5′GP_VK#69	tttctagtagcaactgcaaccggtgtacattctGACATCCAGTTGACACAGCCTGCAT	Vk1-275, Vk1-271, Vk1-225, Vk1-54, Vk1-50, Vk1-46, Vk1-45, Vk1-39, Vk1-35, Vk1-24, Vk1-20, Vk1-14, Vk1-8
	SL_5′GP_VK#70	tttctagtagcaactgcaaccggtgtacattctGACATCCAGCTGACACAGCTTGCAT	Vk1-11
	SL_5′GP_VK#71	tttctagtagcaactgcaaccggtgtacattctGCGTTGCCCTGACACAGTCCCCAGC	Vk6-164
Reverse primers	3′ *Bsi*WI Jk_1_2_3_GP	cagatggtgcagccaccgtacgTTTGATTTCCAGCTTGGTC	JK 1, JK 2, JK 3


For antibody expression, equal amount of heavy and light chain expression vectors containing the paired VH/VK/VL amplicons were transfected into 293F cells with 293fectin transfection reagent (Life Technologies) as previously described (Wang et al., 2016). For a typical transfection reaction, 12.5 μg of each of the VH/VK/VL expression vectors, prepared from 50 ml *E. coli* DH5α cultures, were used to transfect 50 million 293F cells in 50 ml volume. Supernatants were harvested 4 days post-transfection, followed by antibody purification with Protein A Sepharose columns (GE Healthcare). Thus, each guinea pig mAb was expressed as a chimeric mAb with the variable regions (VH/VK or VL) derived from guinea pig and the constant regions from human.

### mAb Binding Analysis by Enzyme-Linked Immunosorbent Assay (ELISA)

The binding specificity of the guinea pig mAbs recovered from single B cells was initially tested with the sorting probe, BG505 SOSIP trimer, and V3 peptides derived from BG505 V3 region (CTRPNNNTRKSIRIGPGQAFYATGDIIGDIRQAHC) and JR-FL (CTRPNNNTRKSIHIGPGRAFYTTGEIIGDIRQAHC) V3 region by ELISA assay. MaxiSorp 96-well plates (Nunc, Thermo Fisher Scientific) were coated with a mouse anti-His tag mAb (R&D Systems, MAB050) at 2 μg/ml in 100 μl of phosphate buffered saline (PBS) at 4°C overnight. After incubating with blocking buffer (PBS containing 5% FBS/2% non-fat milk) for 1 h at 37°C, 2 μg/ml of BG505 SOSIP trimers were added into each well and incubated for 1 h at room temperature. Subsequently, guinea pig mAbs were added in fivefold serial dilutions starting at 50 μg/ml and incubated for 1 h at room temperature. After wash, secondary HRP-conjugated anti-human IgG (Jackson ImmunoResearch) diluted at 1:10,000 in PBS/0.05% Tween 20 was added and incubated for 1 h at room temperature. The signal was developed by adding 100 μl of TMB substrate (Life Technologies) and incubation for 5 min followed by the addition of 100 μl of 1 N sulfuric acid to stop the reactions. The optical density (OD) of each well was measured at 450 nm to quantify binding activity. Between each incubation step, the plates were washed extensively with PBS supplemented with 0.05% Tween 20. The V3 peptides were coated at 2 μg/ml in 100 μl PBS at 4°C overnight, followed by the blocking, binding, washing, and detection procedures as stated above.

### mAb Binding Analysis by BioLayer Interferometry (BLI) Assay

The binding activity and kinetics of selected mAbs to BG505 SOSIP trimer were further assessed by BLI assay via an Octet RED96 system (ForteBio) following the manufacturer’s instruction as previously described (Wang et al., 2016). Octet Analysis version 9.0 software was used for data analysis. The guinea pig mAbs bearing the human IgG constant regions were initially captured by anti-human IgG Fc biosensors, followed by immersing into: (1) analyte wells containing BG505 SOSIP trimer in twofold dilution series in binding buffer (PBS/0.05% Tween 20/0.1% BSA) starting from 1000 to 250 nM to assess association rate (on-rate, *k*_on_), and subsequently (2) wells containing binding buffer to assess dissociation rate (off-rate, *k*_off_). Binding affinity constants (dissociation constant, *K*_D_) were determined as *k*_off_/*k*_on_.

### HIV-1 Neutralization Assays

Antibody/serum neutralization assays were performed in a single round of infection using HIV-1 Env-pseudoviruses and TZM-bl target cells, as previously described (Li et al., 2005). Antibodies (starting at 50 μg/ml) and serum (starting at 10-fold dilution) were diluted in fivefold series to assess neutralization activity. Neutralization curves were fitted by nonlinear regression using a five-parameter hill slope equation. The IC_50_ (or ID_50_) values of each antibody (or serum) were determined as the concentration (or dilution) of antibody (or serum) required to inhibit infection by 50%.

## Results

### Guinea Pig Ag-Specific B Cell Sorting

To analyze B cell response in guinea pigs at the clonal level, we developed this antigen-specific single B cell sorting and mAb isolation method (Figure 1, 2). To evaluate the feasibility of this method, we first sorted HIV-1 Env-specific single B cells by FACS from PBMCs of animal 1567, which displayed potent serum neutralization against the autologous tier 2 virus BG505 and tier 1 isolate ZM109 (Figure 1A,B). We selected animal 1567 for this study as its serum represents the overall virus neutralization profiles of this group, although this serum did not display the highest BG505 neutralization titer within the same group. Class-switched B cells with high level of surface IgG expression profile (IgG^hi^) were distinguished by sequential gating for lymphocytes, single cells, live cells (Aqua blue^-^), and cells with high/low signal for IgG and IgM, respectively (IgG^hi^ IgM^lo^) (Figure 1C). Statistically, from 10 million PBMCs, 4.4 million cells were identified as lymphocytes, and 4.3 million lymphocytes were gated as singlet cells (Figure 1C). Live cells, accounting for 98.3% of the singlet cells were identified with the negative Aqua Blue binding phenotype. 76,545 IgG^hi^ IgM^lo^ B cells were further determined among the live cells at the frequency of 1.82%, based on the fluorescent signals of anti-guinea pig IgM-FITC and anti-guinea pig IgG-Alexa Fluor 594 (Figure 1C). Subsequently, we identified 88 Env-specific B cells (0.11% of IgG^hi^ IgM^lo^ B cells) with the phenotype of IgG^hi^ IgM^lo^/BG505 SOSIP dual^+^ (PE^+^APC^+^) (Figure 1C), which were gated and sorted at single cell density into a 96-well PCR plate for downstream single cell IgG RT-PCR reactions (Table 1).

### Guinea Pig Ig Heavy/Light Chain Amplification

We performed RT-PCR to recover the variable region of heavy/light chain (VH/VK/VL) encoding genes of the sorted Ag-specific guinea pig B cells (Figure 2). The cDNA of each single cell was synthesized by reverse transcription primed by random hexamers. To efficiently amplify Ig genes from cDNA, we developed a semi-nested PCR strategy. All the primers were designed based on the recently annotated guinea pig Ig loci (Guo et al., 2012). From the guinea pig genome sequence annotation study (Guo et al., 2012), 94 VH, 40 DH, and 6 JH gene segments were identified as functional heavy chain genes (Figure 2A). In the Igκ locus, 111 potentially functional Vκ and 3 Jκ genes were determined (Figure 2A). For Igλ, 58 Vλ, and 11 Jλ genes were categorized as potentially functional (Figure 2A). By analyzing the functional gene segment sequences, we designed 5′ forward primers spanning the first 21–25 nt of the framework 1 (FR1) regions in V-gene segments. In total, based on sequence homology, 52, 71, and 36 5′ primers were synthesized for heavy, kappa, and lambda chain amplification, respectively (Figure 2B and Tables 2–4). Two sets of 3′ reverse primers were sequentially used to anneal to the Ig constant regions during PCR reactions (Figure 2C and Tables 2–4). After two rounds of PCR, products were checked on 96-well E Gels. 45/88 (51%) positive wells were observed for the heavy chain amplification, while 36/88 (41%) and 16/88 (18%) positive wells for the lambda and kappa chain amplifications, respectively. From the 88 cells sorted, we successfully recovered 24 paired heavy- and light-chain variable domain genes (Table 1).

All of the amplicons from the second PCR reactions with the expected sizes (Figure 2D) were sequenced and annotated initially by V-Quest through IMGT with human Ig germline database as reference to delineate CDR boundary. Subsequently, we used IgBLAST to determine the closest functional V, D, and J segments using in-house guinea pig germline database derived from the work of Guo et al. (2012).

### Guinea Pig mAb Cloning and Expression

Based on the V and J segments assigned with IgBLAST, the corresponding cloning PCR primers for each mAb were chosen from the seamless cloning primer sets shown in Tables 5–7. Cloning PCR products were further inserted into expression vectors by GeneArt seamless cloning and assembly kit, followed by sequencing verification. By co-transfection of heavy- and light-chain expression vectors into 293F cells, 20 out of 24 (83%) chimeric mAbs were efficiently expressed and purified from cell culture supernatants. Genetic analysis grouped them into nine clonal lineages, with most lineages consisting single member while three lineages (#1, #5, and #7) containing multiple clonal members (Figure 2E and Table 8). Clonal dominance is notable: one clonal lineage #5 (Figure 2E and Table 8) consisting of 10 clonal members predominantly accounts for 50% of the total sorted B cells. Somatic hypermutation (SHM) levels (Mut %) of the sorted B cells were calculated as percentage of nucleotide sequence divergence from the germline V gene sequences (Table 8). We found moderate level of SHM for the VH and VK/VL of the sorted B cells, ranging from 2.9 to 11.5%, and 0.7 to 12%, respectively (Figure 2E and Table 8), consistent with the SHM level of Env-specific mAbs elicited by immunization in rhesus monkeys reported previously (Wang et al., 2016).

**Table 8:** Genetic and binding specificity analysis of guinea pig mAb variable region sequences^‡^.

			VDJ segments_CDRH3			VJ segments_CDRL3			Binding specificity
	mAb	Lineage #	length (AA)	VH Mut (%)	CDRH3 AA seq	length (AA)	VL Mut (%)	CDRL3 AA seq	(ELISA/BLI)
nAbs	CP2	1	VH3-197_D6_JH4_CDR3_17	7.5	TRNLLLSGVESTGAFDV	VL3-41_JL_3_CDR3_13	2.0	AITLGSGSIFQSV	Env (V3)
	CP94	1	VH3-197_D6_JH4_CDR3_17	8.4	TRNLLLSGVESTGAFDV	VL3-41_JL_3_CDR3_13	2.0	AITLGSGSIFQSV	Env (V3)
	CP6	2	VH3-197_D18_JH4_CDR3_12	11.0	AKNKGETASFDV	VL3-41_JL_1_CDR3_13	6.0	SIARVSGNNIQWV	Env (V3)
	CP10	3	VH3-197_D3_JH4_CDR3_15	9.1	AKNGDTSNGETTPDV	VL3-41_JL_1_CDR3_13	6.0	TVAHVSGNAFQWV	Env (V3)
	CP67	4	VH3-197_D17_JH4_CDR3_17	7.3	AKNLLLSEATSTGAFDV	VL4-82_JL_1_CDR3_9	12.0	AVGHSAGWV	Env (V3)
Non- nAbs	CP3	5	VH3-183_D15_JH4_CDR3_18	8.0	TRGPYYRWGTWSLYYFDI	VL4-82_JL_1_CDR3_9	6.6	AVGYNTGWV	Env, unknown
	CP7	5	VH3-183_D15_JH4_CDR3_18	11.2	ARAPYYKWGTWSLYYFDI	VL4-82_JL_1_CDR3_9	8.4	AVGHSAGWV	Env, unknown
	CP13	5	VH3-183_D15_JH4_CDR3_18	6.9	ARGAYYRWGTWSLYYFDV	VL4-82_JL_1_CDR3_9	5.1	AVGYSAGWV	Env, unknown
	CP37	5	VH3-183_D15_JH4_CDR3_18	7.7	ARGAYYKWGTWTLYYFDL	VL4-82_JL_1_CDR3_9	7.6	AVGHSAGWV	Env (unknown)
	CP53	5	VH3-183_D15_JH4_CDR3_18	11.4	ARAPYYKWGTWSLYYFDI	VL4-82_JL_1_CDR3_9	7.6	AVGHSAGWV	Env (unknown)
	CP61	5	VH3-183_D15_JH4_CDR3_18	7.7	ARGPYYRWGTWSLYYFDI	VL4-82_JL_1_CDR3_9	5.8	AVGYSAGWV	^∗^Env (unknown)
	CP62	5	VH3-183_D15_JH4_CDR3_18	11.5	ARAPYYKWGTWSLYYFDI	VL4-82_JL_1_CDR3_9	9.8	AVGHSAGWV	^∗^Env (unknown)
	CP73	5	VH3-183_D15_JH4_CDR3_18	11.1	ARGPYYRWGTWSLYYFDI	VL4-82_JL_1_CDR3_9	7.6	AVGYSAGWV	Env (unknown)
	CP91	5	VH3-183_D15_JH4_CDR3_18	7.6	ARGPYYKWGTWSLYYFDI	VL4-82_JL_1_CDR3_9	7.3	AVGYSAGWV	^∗^Env (unknown)
	CP92	5	VH3-183_D15_JH4_CDR3_18	7.0	ARGAYYKWGTWTLYYFDL	VL4-82_JL_1_CDR3_9	8.0	AVGYSAGWI	Env (unknown)
	CP68	6	VH3-183_D15_JH2_CDR3_18	8.3	QEGSYYKWGMGMTSNHHA	VL4-82_JL_1_CDR3_9	6.6	AVGYSAGWV	Env (unknown)
	CP58	7	VH3-230_D31_JH2_CDR3_7	5.1	TTTGLTY	Vk1-201_Jk2_CDR3_9	3.6	WQYDKLPLT	Env (unknown)
	CP63	7	VH3-230_D17_JH2_CDR3_7	2.9	TTTGLGY	Vk1-201_Jk2_CDR3_9	3.5	WQYDKLPLT	Env (unknown)
	CP66	8	VH3-139_D27_JH4_CDR3_7	2.9	ARMVVDV	Vk4-83_Jk2_CDR3_9	0.7	MQDYNPPYT	Env (unknown)
	CP82	9	VH3-80_D31_JH4_CDR3_17	3.7	ARDGWEEYMWGGSFLDL	Vk4-28_Jk3_CDR3_11	1.9	LQYYDFPNT	Env (unknown)


*^‡^Heavy- and light-chain sequences of mAbs cloned were analyzed by IgBLAST and assigned to the closest V, D, and J germline genes. Clonal lineage criteria are shown in Figure 2. Somatic hypermutation level (Mut %) were calculated as percentage of nucleotide sequence divergence from germline V gene sequences. ^∗^ELISA/BLI negative. Specificity inferred by clonal lineage analysis, e.g., belonging to the same lineage with BG505 SOSIP binding clones.*

### Guinea Pig mAb Characterization

**FIGURE 3:**
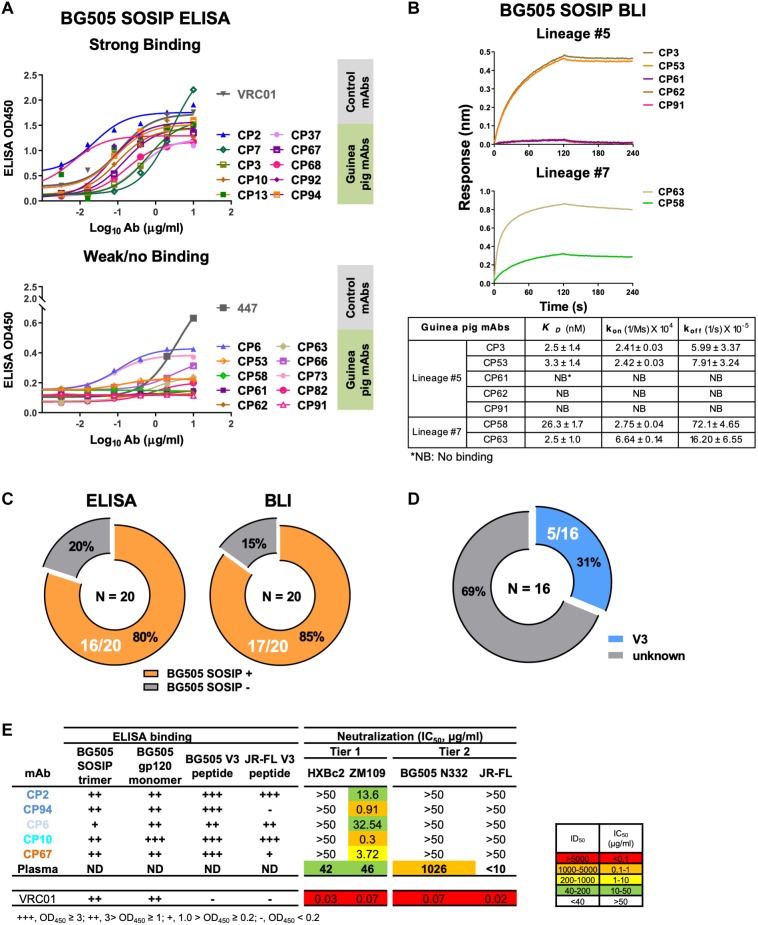
Binding specificity and neutralization profile of guinea pig mAbs isolated from PBMCs. **(A)** Reactivity of guinea pig mAbs to BG505 SOSIP trimer by ELISA assay. Strong binding (OD450 >0.5); weak/no binding (weak-binding, 0.2<OD450 <0.5; no binding, OD450 <0.2). Data were duplicated, with the mean of OD450 shown. **(B)** Reactivity of selected guinea pig mAbs to BG505 SOSIP trimer by BLI assay. (Upper) BLI binding curves of mAbs from clonal lineages #5 and #7. mAbs were captured by anti-human IgG Fc biosensors, followed by interaction with BG505 SOSIP trimer in twofold dilution series starting from 1000 to 250 nM, with the curves of 1000 nM shown. (Lower) mAb-BG505 SOSIP trimer binding kinetic parameters, shown as measured value ± standard error. *k*_on_, on-rate or association rate; *k*_off_, off-rate or dissociation rate; *K*_D_, binding affinity constants (dissociation constant), determined as *k*_off_/*k*_on_. **(C)** Summary of BG505 SOSIP trimer reactivity by ELISA and BLI. Of 20 mAbs expressed (left) 16 (80%) and (right) 17 (85%) show detectable binding activity to BG505 SOSIP trimers by ELISA and BLI assay, respectively. **(D)** Pie chart showing the binding specificity of the 16 BG505 SOSIP-reactive mAbs isolated from guinea pig 1567 by ELISA assay. V3-reactivity was assessed by positive reactivity (OD450 >0.2) with peptides derived from V3 regions of HIV isolates BG505 or JR-FL. **(E)** Neutralization potency (IC_50_, μg/mL) of isolated mAbs against Env-pseudotyped viruses. mAbs are color-coded to differentiate corresponding clonal lineage. Animal 1567 plasma neutralization titers (ID_50_) against HXBc2, ZM109, BG505 N332 (autologous virus corresponding to the immunogen, BG505 SOSIP, which contains N332 glycan), and JR-FL pseudoviruses were shown for comparison. ID_50_ are values of the plasma reciprocal dilution factors at which 50% inhibition of infection are achieved in the assay. The background neutralization ID_50_ titer threshold is set as <10. ND, not determined. ELISA binding to BG505 SOSIP trimer, BG505 gp120 monomer, BG505 V3 and JR-FL V3 peptide are also shown. Broadly neutralizing mAb, VRC01 (CD4bs epitope specific) is shown as control. Data are duplicated with the mean of IC_50_/ID_50_ shown.

Since we sorted HIV-1 Env-specific B cells for Ig encoding gene analysis and mAb cloning, we further verified the mAb binding specificity by ELISA binding assays, with BG505 SOSIP pre-coated on ELISA plates and mAbs serving as analytes. We found that 16/20 (80%) of the guinea pig mAbs recognized the antigen probe, BG505 SOSIP trimer by ELISA assay (Figure 3A and Tables 1, 8). The predominant clonal lineage #5, consists seven members that bind BG505 SOSIP well (Figure 3A and Table 8) and three members (CP61, 62, and 91) showing negligible BG505 SOSIP binding assayed by ELISA (Figure 3A and Table 8). This observation demonstrates the heterogeneity of affinity for antigen between clonal members within the same clonal lineage.

We subsequently used BLI assay to characterize the antigen binding affinity of selected guinea pig mAbs from lineages #5 and #7, which consist mAb members with negative antigen binding activity assessed by ELISA (Figure 3B and Table 8). In this BLI assay, the mAbs bearing the human Fc portion were initially captured to the anti-human IgG Fc biosensors followed by the association reaction with BG505 SOSIP analytes, which assesses the binding reaction in an orientation different from that in the ELISA assay. Thus, the BLI assay result would complement the ELISA measurement, especially for epitopes sensitive to antigen pre-coating. We selected a few members (Table 8) from the clonal lineage #5 with different ELISA binding profiles, including CP3 (high binding), CP53 (weak binding), and CP61, 62, and 91 (no binding), as well as CP58 (no ELISA binding) in clonal lineage #7 for BLI assay. We found that consistent with the ELISA assay, CP3 and CP53 showed decent binding signal for BG505 SOSIP (Figure 3B), while no binding signal was observed for CP61, 62, and 91. Interestingly, CP53 showed affinity (dissociation constant *K*_D_ approximately 2 nM) for BG505 SOIP equivalent to CP3 in this BLI assay, while its binding to BG505 SOSIP was weaker than CP3 in ELISA assay (Figure 3A). For mAbs from lineage #7, CP58 and CP63 displayed moderate (*K*_D_ = 26 nM) and strong (*K*_D_ = 2.5 nM) binding affinity for BG505 SOSIP (Figure 3B), respectively, while they showed no and weak ELISA binding to BG505 SOSIP, separately. The overall improved antigen binding profiles of CP58 and CP63 from lineage #7 by BLI assay indicate that their cognate epitope on the BG505 SOSIP trimer is sensitive to the antigen pre-coating in the ELISA assay. Our results suggests that the results of ELISA and BLI binding assays corroborate with each other, while the sensitivity of BLI assay is often higher than ELISA. Based on BLI assay, 85% (17/20) of the guinea pig mAbs recognized the antigen probe, BG505 SOSIP trimer (Figure 3C and Tables 1, 8). Therefore, BLI assay could be used to complement ELISA for assessing Ab-Antigen binding (Figure 3C).

We then estimated the precision of our Ag-specific class-switched B cell sorting and Ig cloning method by consolidating the results of Ag-binding assays (ELISA and BLI) and genetic analysis. Since B cells derived from the same clonal lineage share similar Ag recognition determinants, we used clonal lineage information of the sorted mAbs to infer the Ag-binding specificity of mAbs with inappreciable Ag ELISA/BLI binding phenotypes. For example, CP61, 62, and 91 from clonal lineage #5 barely bound BG505 SOSIP (Figure 3A,B). However, their related mAb clones such as CP3 and CP92 from lineage #5 showed strong binding with BG505 SOSIP trimer by ELISA and/or BLI (Figure 3A,B). Thus, we inferred the Ag specificity of CP61, 62, and 91 to be the same with CP3 and CP92 (Table 8). Such mAbs showing negative antigen binding phenotypes in ELISA/BLI assays possess low affinity for BG505 SOSIP (Figure 3B). However, in the FACS sorting procedure, streptavidin-PE or -APC conjugates were premixed with the biotin-labeled BG505 SOSIP trimers to form high-order sorting probe complex with elevated binding valence, which could presumably facilitate the binding and isolation of B cells with low affinity B cell receptors encoding the above stated mAbs. Thus, in addition to functional antigen-binding assays, clonal lineage analysis is informative for determining the antigen-binding specificity of low affinity mAbs. To the end, we concluded that all the 20 mAbs derived from the Ag-selection based sorting/cloning study were Ag-specific (Tables 1, 8), with various affinities for the cognate Ag (Figure 3B), belonging to nine clonal lineages (Figure 2E and Table 8). Thus, the sorting and cloning method is feasible for guinea pig Ag-specific B cell analysis, with virtually 100% precision (Table 1).

To map the binding epitopes of the mAbs, we tested their reactivity against V3 peptides derived from the Envs of BG505 and JR-FL, since the V3 region is one of the immunodominant epitopes of HIV Env shown by previous studies (Phad et al., 2015; Wang et al., 2017). We found that five mAbs including CP2, CP6, CP10, CP67, and CP94 bound the autologous BG505 V3 peptide well (Figure 3D,E), while three of them (CP2 and CP94 from clonal lineage #1, and CP10 from clonal lineage #3) bound V3 peptide derived from isolate JR-FL with high binding activity (Figure 3E), suggesting the prominent immunogenicity of the BG505 SOSIP V3 region in this study. The substantial frequency of V3-reactive mAbs resulted from this immunization is consistent with the notion that V3 region is still a prominent immunogenic element of the current generation of HIV-1 Env trimer immunogen such as BG505 SOSIP.

To characterize the function of the isolated mAbs further, we analyzed their neutralization capacity via the TZM-bl assay utilizing a panel of pseudotyped HIV-1 viruses. Five mAbs from four clonal lineages neutralized the tier 1 virus ZM109, which is consistent with the polyclonal plasma neutralization capacity against the same virus (Figure 3E). All the tier 1 virus neutralizing antibodies recognize V3 region (Figure 3E). It is notable that none of the cloned mAbs from this study neutralizes the autologous virus BG505 N332 as the plasma does (Figure 3E). BG505 SOSIP trimer contains the conserved V3 glycan N332 (Sanders et al., 2013). Therefore, virus BG505 N332 is used as the virus for assessing mAb and plasma autologous neutralization activities.

## Discussion

Small animal models are typically utilized for initial preclinical evaluation of vaccine candidates *in vivo*. The guinea pig model, compared to mouse, is more immunologically similar to human and has an adequate amount of blood volume for initial immunological analysis (Padilla-Carlin et al., 2008). Some recent studies have used guinea pig model to evaluate the immunogenicity of HIV Env-based immunogen candidates and immunization strategies (Nkolola et al., 2010; Feng et al., 2016; Zhou et al., 2017). However, characterization of B cell response in these studies is still limited to the polyclonal level. Besides serum neutralization assay and epitope mapping, a high-resolution strategy to delineate B cell response is desirable, as it will substantially aid vaccine evaluation and vaccine design.

There are two major technique hurdles obstructing single B cell analysis in guinea pigs: (i) the lack of sophisticated B cell surface marker antibody panels for identifying guinea pig memory B cells, in contrast to mouse, macaque, and human (Tiller et al., 2008, 2009; Sundling et al., 2012), and (ii) the unavailability of primer sets for amplifying guinea pig IGH- and light-chain encoding genes. In this study, we took advantage of the expression of B cell receptors (BCR) on the surface of IgG^hi^ IgM^lo^ B cells, which renders the corresponding B cells recognizable by antigens and anti-IgG secondary antibodies. Using FACS sorting technique with fluorochrome-conjugated antigen and anti-guinea pig IgG secondary antibody cocktails, we successfully isolated antigen-specific guinea pig class-switched IgG^hi^ B cells for further analysis. The combination of antigen and anti-guinea pig IgG secondary antibody is sufficient to capture antigen-specific B cells, as demonstrated that >85% mAbs cloned from the sorted B cells are specifically reactive to the sorting antigen probe. In addition, we designed a set of guinea pig Ig gene-specific primers based on recently annotated guinea pig Ig gene cluster mapping (Guo et al., 2012), by using a pool of 5′ forward primers derived from the framework 1 of guinea pig heavy- and light-chain variable domains, and 3′ reverse primers annealing to the constant regions of guinea pig heavy/light chains. We recovered guinea pig Ig sequences by a semi-nested PCR strategy similar to the strategies proposed for mouse (Tiller et al., 2009), rabbit (Starkie et al., 2016), macaque (Sundling et al., 2012), and human (Tiller et al., 2008). Approximately 50–60% heavy and light chain recovery efficiency was observed, while 27% of sorted cells have paired heavy and light chain amplicons for further functional analysis. In complement to a previous elegant study focused on enriching plasmablast cells (PCs), which contain abundant endoplasmic reticulum (ER) with ER-specific fluorescent dye to recover Ig encoding genes (Kurosawa et al., 2012), our platform is applicable to analyze various class-switched B cell populations including memory B cells and a subset of PCs, which remain expression of cell surface-bound IgGs.

We isolated five mAbs from four clonal lineages encoding tier 1 virus neutralizing antibodies targeting the V3 crown of the HIV-1 Env (Figure 3E and Table 8). The failure of these V3-specific mAbs to neutralize tier 2 viruses (Figure 3E) is consistent with the notion that the V3 crown is mostly occluded by other structural elements such as the V1V2 loops and glycans on the static (closed) HIV Env trimers of primary isolates (e.g., tier 2 viruses such as BG505) (Julien et al., 2013; Lyumkis et al., 2013; Kwon et al., 2015). However, these V3-specific mAbs are able to access the V3 crown on the tier 1 virus Env spikes, which sample more opened configurations than tier 2 viruses (Zolla-Pazner et al., 2016; Wang et al., 2017), to mediate tier 1 virus neutralization. In addition, we found that these V3-specific mAbs all use VH3-197/JH4 and mostly VL3-41 segments (Table 8). Moreover, we identified a predominant clonal lineage consisting of 10 clonal members (Lineage #5, Table 8) with the usage of VH3-183_D15_JH4 and VL4-82_JL1 gene segments (Table 8), which accounts for 50% of the well-expressed mAbs derived from the Ag-specific IgG^hi^ IgM^lo^ B cells with paired heavy/light chains. The observed skewed Ig gene segment usage highlights the immunodominance of certain B cell lineages in the Env-specific B cell repertoire following BG505 SOSIP trimer immunization.

Of note, none of the mAbs isolated from the PBMCs (∼10 million) of animal 1567 in this study displayed neutralization against the autologous tier 2 virus, BG505, which is consistent with the observed relatively low frequency of Env-specific B cells (0.11% in class-switched B cells) in the PBMC compartment of this animal (Figure 1C and Table 1). Sampling lymphoid tissues including spleen and lymph nodes where B cell germinal center activation primarily occurs (Victora and Nussenzweig, 2012) may lead to capturing the antigen-specific IgG^hi^ B cells at higher frequency. In a related study (Lei et al., 2019) using similar BG505 SOSIP sorting probes, we found that about 3% of class-switched IgG^hi^ B cells are antigen-specific in splenocytes, which is 30-fold higher than that in PBMCs (Figure 1C). Subsequently, using splenic B cells and a more selective sorting strategy, we were able to capture the BG505 Env-specific B cells, which encode the antibodies recapitulating the autologous serum neutralization capacity against the BG505 virus with this cloning platform (Lei et al., 2019). Therefore, this method could be further applied to examine Ag-specific B cell repertoire from different tissue compartments in future studies.

This method enables us sampling 27% Ag-specific B cells with paired heavy- and light-chain from 88 Ag-reactive single IgG^hi^ IgM^lo^ B cells (out of 10 million PBMCs) for functionality analysis, defining clonal lineage relationship between sorted B cells, and delineating epitope binding specificity of selected mAb clones (Figure 3 and Table 8). Our result demonstrates the essential efficiency and feasibility of this platform for guinea pig single B cell analysis. The relatively low frequency of sorted cells with paired heavy and light chain amplicons may be caused by Ig gene polymorphisms (Corcoran et al., 2016). We anticipate that with the improvement in guinea pig Ig primer set design in the future, by primer optimization informed by individualized V gene sequencing (Corcoran et al., 2016), this platform can be more efficient and comparable to previous methods developed for mouse, macaque, and human (Tiller et al., 2009; Wu et al., 2010; Zhao et al., 2017).

With the method developed herein, specific antibody lineages responsible for neutralization activity can be identified and characterized from vaccinated animals. In addition, the primers designed for guinea pig single cell RT-PCR can be applied for next-generation Ig sequencing library preparation to interrogate B cell clonal lineage evolution during immunization. All the information gleaned from these analyses will contribute to a better understanding of immune response quality and detailed specificities to inform future vaccine design. Furthermore, this method enables the isolation of Ag-specific mAbs for developing therapeutic reagents in guinea pig model. Thus, the platform described here will clearly benefit future B cell response analyses at both the clonal and repertoire levels, which helps to facilitate future efforts in immune response characterization as well as genetic and functional interpretation of the guinea pig model for applicable infectious diseases, including but not limited to HIV-1.

## Ethics Statement

The animal study was carried out at Covance with the protocol approved by the Covance Institutional Animal Care and Use Committee (IACUC), with IACUC protocol #0138–14.

## Author Contributions

YL and LL conceived the study. LL, JS, YW, and C-IC developed the methodology. LL, KT, YW, JS, C-IC, YX, RW, and YL investigated the results. YL and RW supervised the study. YL and C-IC administered the project. YL and RW acquired funding. LL drafted the manuscript. YL, LL, and JS reviewed and edited the manuscript.

## Conflict of Interest Statement

The authors declare that the research was conducted in the absence of any commercial or financial relationships that could be construed as a potential conflict of interest. Part of this work took place at Covance as a fee-for-service. Covance had no involvement with the study.

## Footnotes

1 http://www.ensembl.org

## Abbreviations

bNAbs: broadly neutralizing antibodies
BLI: BioLayer Interferometry
CDR3: complementarity-determining region
D: diversity
ELISA: enzyme-linked immunosorbent assay
FACs: fluorescence-activated cell sorting
Ig: immunoglobulin
IGH: Ig heavy
IGL: Ig lambda
IGK: Ig kappa
J: joining
mAb: monoclonal antibodies
PBMCs: peripheral blood mononuclear cells
V: variable
